# Polyarthritis spectrum disorders managed by ayurvedic formulation arthocon: A clinical case series

**DOI:** 10.6026/973206300211901

**Published:** 2025-07-31

**Authors:** Danish Javed, Rashmi Verma, Sana Anwar, Ranjana Pandey

**Affiliations:** 1 Department of AYUSH, All India Institute of Medical Sciences (AIIMS), Bhopal, India; 2 Department of Trauma and Emergency Medicine, All India Institute of Medical Sciences, Bhopal, Madhya Pradesh, India; 3 Department of Community and Family Medicine, All India Institute of Medical Sciences (AIIMS), Bhopal, India

**Keywords:** Polyarthritis, joint disorders, inflammation

## Abstract

Polyarthritis encompasses a broad range of joint disorders, including autoimmune, degenerative and crystal-induced types. Conventional treatments often involve long-term NSAIDs or immunosuppressants, with significant side effects. In this case series, fifteen adult patients (age 29 to 63 years; both male and female) with confirmed diagnoses of RA, PsA, SLE, AS, OA and polyarticular gout were enrolled and treated with Arthocon^TM^ Capsule (500 mg BID) and topical Arthocon^TM^ Oil for 12 weeks. Mean joint count decreased from 11.5 to 3.8, swelling index from 8.9 to 2.1 and VAS pain scores from 7.3 to 2.8. Disease activity scores improved significantly: DAS28 (mean reduction 2.9), PASDAS (mean reduction 3.2), SLEDAI (mean reduction 6.0), BASDAI (mean reduction 3.1), WOMAC (mean reduction 34.7) and SUA in gout patients (mean reduction 1.9 mg/dL) without any major adverse effects. The Ayurvedic combination of Arthocon^TM^ Capsule and Oil demonstrated promising results across different arthritis types, suggesting its potential as a safe and effective integrative approach in the management of chronic joint disorders.

## Background:

Arthritis represents a major global health challenge, affecting over 350 million people worldwide, with significant implications for quality of life, work productivity and healthcare expenditure [1]. Globally, musculoskeletal disorders are the leading contributors to disability-adjusted life years (DALYs), especially in the aging population [2]. The term "polyarthritis" refers to inflammation involving five or more joints and encompasses a wide clinical spectrum of disorders ranging from autoimmune diseases such as Rheumatoid Arthritis (RA), Psoriatic Arthritis (PsA) and Systemic Lupus Erythematosus (SLE), to Seronegative Spondylo-arthropathies like Ankylosing Spondylitis (AS), as well as degenerative conditions such as Osteoarthritis (OA) and metabolic arthropathies like Polyarticular Gout [3]. Each of these conditions has a distinct pathophysiological mechanism. RA is a chronic, systemic autoimmune disorder characterized by synovial hyperplasia, pannus formation and joint erosion, primarily driven by pro-inflammatory cytokines including TNF-α, IL-1β and IL-6 [4]. PsA is associated with aberrant immune activation in the setting of psoriasis and exhibits enthesitis, dactylitis and joint erosion, with involvement of both innate and adaptive immune pathways, particularly IL-17/IL-23 axis [5]. SLE, a systemic autoimmune disorder, involves immune complex deposition and complement activation, often presenting with non-erosive polyarthritis along with multi-organ involvement [6]. AS, part of the spondylo-arthropathy group, is characterized by axial skeletal inflammation and sacroiliitis, typically associated with HLA-B27 positivity and overexpression of IL-17A [7]. OA represents a non-inflammatory degenerative disease in its initial stages, marked by cartilage loss, sub-chondral bone remodelling and low-grade synovial inflammation, driven by matrix metalloproteinases (MMPs) and oxidative stress [8]. Polyarticular Gout, though classically monoarticular, may involve multiple joints in chronic stages due to monosodium urate (MSU) crystal deposition and intense neutrophilic infiltration [9].

The conventional management of these diseases includes NSAIDs, corticosteroids, DMARDs (*e.g.*, methotrexate, sulfasalazine) and biological agents (*e.g.*, anti-TNF, IL-6 inhibitors). While these agents have transformed disease outcomes, their long-term use is associated with notable side effects such as gastrointestinal ulceration, hepatotoxicity, renal dysfunction, immunosuppression and increased risk of infections [10, 11]. Furthermore, not all patients achieve sustained remission, underscoring the need for safe, accessible and integrative treatment approaches. There is a growing interest in complementary and alternative medicine (CAM), particularly Ayurveda, for safer, long-term management of chronic arthropathies [12]. Ayurvedic medicine conceptualizes joint disorders under various classifications such as Amavata, Vatarakta, Sandhivata and Kushtha-janya Sandhigata Vata, depending on doshic imbalance and clinical features [13]. Notably, herbs like *Boswellia serrata* (Shallaki), Commiphora mukul (Guggulu), *Withania somnifera* (Ashwagandha) and Tinospora cordifolia (Guduchi) have shown promising activity against key inflammatory pathways. Shallaki (*Boswellia serrata*) has demonstrated COX-2 inhibition, suppression of 5-lipoxygenase and reduction in leukotriene production, leading to pain relief and cartilage protection [14]. Guggulu (Commiphora mukul) contains guggulsterones, known for their anti-inflammatory, antioxidant and lipid-lowering effects [15]. Ashwagandha (*Withania somnifera*) exhibits immunomodulatory, anti-stress and cortisol-lowering effects, useful in autoimmune conditions [16]. Guduchi (Tinospora cordifolia) recognized for its TNF-α suppressing, macrophage-modulating and antioxidant activities [17]. Therefore, it is of interest to report the application of Ayurvedic formulation Arthocon Capsule and Arthocon Oil across a diverse group of arthritic patients in this case series.

## Methodology:

## Study design:

Prospective open-label case series.

## Participants:

Fifteen patients (aged 29 to 63 years; both males and females) with a confirmed diagnosis of RA, PsA, AS, SLE, OA, or Polyarticular Gout based on established diagnostic criteria (ACR/EULAR 2010 for RA, CASPAR for PsA, SLICC for SLE, Modified New York Criteria for AS, ACR 1986 for Gout and ACR 2010 for OA).

## Inclusion criteria:

Adults aged 18-65 years with clinically active arthritis, VAS >6 and willingness to undergo Ayurvedic treatment.

## Exclusion criteria:

Recent use of biologics, pregnancy/lactation, uncontrolled systemic illness

## Intervention:

[1] Arthocon Capsule: 500 mg orally, with water, twice daily after meals.

[2] Arthocon Oil: Topical application twice daily with gentle massage over affected joints.

[3] Duration: 12 weeks

Arthocon^TM^ Capsule, manufactured and marketed by Sushila Herbal, is an Ayurvedic proprietary formulation containing Herbo-mineral ingredients like Ekangveer Ras, Sutshekhar Ras, MahaVaat Vidhvans Ras, SameerPaanag Ras, Khurasani Ajwain (*Hyoscyamus niger*), Punarnava (*Boerhavia diffusa*), Nirgundi (*Vitex negundo*), Shallaki (*Boswellia serrata*), Ashwagandha (*Withania somnifera*) and Erand Moola (*Ricinus communis*) and has demonstrated anti-inflammatory, analgesic and immunomodulatory properties in clinical practice. Arthocon^TM^ Oil, from same manufacturing unit, is a polyherbal Ayurvedic proprietary formulation, contains Mahanarayan oil, Wintergreen oil, Mentha oil, Gaultheria oil, Kapoor oil (*Cinnamomum camphora*), Allium oil (*Allium sativum*), Clove oil, (*Syzygium aromaticum*), Capsicum oil (*Capsicum annum*) and Sesame oil (*Sesamum indicum*).

## Outcome measures:

[1] Primary: Joint count, swelling index, VAS pain score

[2] Secondary: ESR, CRP, disease-specific indices (DAS28, PASDAS, BASDAI, SLEDAI, WOMAC, SUA)

## Statistical analysis:

Descriptive statistics were used to summarize results. Paired t-test was applied for pre- and post-treatment comparisons.

## Results:

[1] Pain reduction: VAS scores showed a mean reduction from 7.3 ± 0.6 to 2.8 ± 0.4 (p < 0.01).

[2] Joint count: Mean reduced from 11.5 to 3.8.

[3] Swelling index: Reduced from a mean of 8.9 to 2.1.

[4] ESR and CRP: Both markers reduced significantly across all cases, reflecting reduced systemic inflammation.

[5] Disease activity scores: DAS28, PASDAS, SLEDAI, BASDAI and WOMAC scores showed a clinically meaningful improvement, indicating effective disease control.

[6] No major adverse effects: Were reported during the 12-week intervention with Arthocon Capsule and Oil.

## Discussion:

This case series evaluates the clinical effectiveness and safety of the Ayurvedic formulation Arthocon Capsule and Arthocon Oil in a heterogeneous group of patients with polyarthritis, encompassing autoimmune, degenerative and crystal-induced arthropathies (Table 1 - see PDF). The findings suggest that this herbo-mineral combination has a broad-spectrum therapeutic profile, offering significant symptom relief and systemic inflammation control without major adverse events over a 12-week treatment period. The observed reduction in mean joint count (from 11.5 to 3.8), swelling index (from 8.9 to 2.1) and VAS pain scores (from 7.3 to 2.8) is clinically meaningful and statistically significant (p < 0.001), indicating tangible symptomatic improvement (Table 2 - see PDF and Table 3 - see PDF). These outcomes align with prior preclinical and clinical studies evaluating the efficacy of key constituents such as *Boswellia serrata*, Commiphora mukul and *Withania somnifera*, which have shown strong anti-inflammatory, analgesic and immunomodulatory properties. Specifically, in rheumatoid arthritis (RA), patients demonstrated an average reduction in DAS28 score from 6.2 to 3.3, a change consistent with transitioning from high to low disease activity. The improvement mirrors findings from trials investigating Boswellia serrata, which inhibits 5-lipoxygenase and leukotriene synthesis, thereby reducing synovial inflammation and joint degradation [18]. Similarly, Guggulu (Commiphora mukul) has been reported to exert anti-inflammatory effects through downregulation of NF-κB and suppression of COX-2 expression, both central in RA pathogenesis [19]. Previous randomized controlled studies are also suggestive that Commiphora mukul, *Boswellia serrata*, and *Withania somnifera* reduce RA factor significantly in rheumatoid arthritis patients as well as reduction s seen in WOMAC score in cases of osteoarthritis [20]. Psoriatic arthritis (PsA) patients experienced substantial improvements in PASDAS scores (mean reduction from 5.9 to 2.4), consistent with reduced dactylitis, enthesitis and systemic inflammation. The immunological basis of PsA, involving IL-23/IL-17 axis and Th17 polarization, has shown responsiveness to herbal immunomodulators such as Ashwagandha and Tinospora cordifolia, which have demonstrated inhibition of pro-inflammatory cytokines (IL-6, TNF-α) and macrophage activation [21, 22]. In systemic lupus erythematosus (SLE), SLEDAI scores dropped from a mean of 9.5 to 3.5, indicative of improved disease control. While Ayurvedic literature classifies SLE under "Amavata" or "Vatarakta," modern interpretation supports immune-regulatory herbs like Guduchi and Ashwagandha in suppressing autoantibody generation and complement activation. These herbs modulate innate immunity, promote antioxidant defense and attenuate T-cell hyperactivation, which are central to SLE pathogenesis [23]. Patients with ankylosing spondylitis (AS) demonstrate notable reductions in BASDAI scores (mean from 5.8 to 2.7), suggesting improved spinal mobility and reduced axial stiffness. The role of Shallaki, Erand Moola and Nirgundi in mitigating sacroiliac inflammation and enhancing musculoskeletal flexibility has been corroborated by prior observational studies in spondyloarthropathies. Osteoarthritis (OA) patients showed substantial improvements in WOMAC scores (mean reduction from 71.6 to 36.9), reflecting improved joint function and reduced stiffness. OA, often labeled as "Sandhivata" in Ayurveda, is approached with formulations aimed at enhancing synovial lubrication and reducing oxidative cartilage damage. Sesame oil-based topical formulations, as used in Arthocon Oil, may facilitate transdermal drug delivery and provide local anti-nociceptive effects. In gout, serum uric acid (SUA) decreased by an average of 1.9 mg/dL, accompanied by joint pain and swelling relief (Table 4 - see PDF). Ayurvedic herbs such as Punarnava and Eranda possess documented uricosuric and anti-inflammatory effects, which may underlie this therapeutic benefit [24]. Importantly, inflammatory markers ESR and CRP demonstrated consistent and statistically significant reductions across all diagnostic categories, supporting systemic immunomodulation. Notably, no major adverse events were reported, underscoring the safety profile of the intervention, even in autoimmune conditions where immune suppression can pose risks. Nonetheless, this study has limitations. The small sample size (n=15), absence of a comparator group and open-label design limit the generalizability and causal inference. The subjective nature of pain reporting (VAS) and potential placebo effects must also be acknowledged. However, the use of validated disease activity indices and biochemical markers provides a level of objectivity and clinical relevance. In summary, the polyherbal and herbo-mineral composition of Arthocon appears to address multiple pathophysiological pathways immune dysregulation, cytokine overexpression, oxidative stress and cartilage degradation across diverse arthritic conditions. These findings warrant further exploration through larger, randomized, placebo-controlled trials to confirm efficacy and delineate mechanisms of action.

## Conclusion:

This case series shows the promising clinical utility of the Ayurvedic formulation Arthocon Capsule and Oil in the management of diverse polyarthritis spectrum disorders. The absence of significant adverse effects further supports its potential role as a safe integrative therapy. Future randomized controlled trials with larger cohorts and mechanistic studies are essential to establish definitive efficacy, safety and the pharmacological pathways involved.

## Funding:

Nil

## References

[1] Eakin G.S (2017). Dela J Public Health..

[2] Safiri S (2021). Arthritis Rheumatol..

[3] Alpay-Kanitez N (2018). Eur J Rheumatol..

[4] Guo Q (2018). Bone Res..

[5] Azuaga A.B (2023). Int J Mol Sci..

[6] Chia J.E (2025). Int J Rheum Dis..

[7] McGonagle D.G (2019). Ann Rheum Dis..

[8] Sanchez-Lopez E (2022). Nat Rev Rheumatol..

[9] Martillo M.A (2014). Curr Rheumatol Rep..

[10] Shams S (2021). Front Pharmacol..

[11] https://www.ncbi.nlm.nih.gov/books/NBK507863/.

[12] Khan M.U (2016). J Clin Diagn Res..

[13] Akhtar B (2010). Ayu..

[14] Shin M.R (2022). Evid Based Complement Alternat Med..

[15] Deng R (2007). Cardiovasc Drug Rev..

[16] Mikulska P (2023). Pharmaceutics..

[17] Gupta A (2024). Heliyon..

[18] Siddiqui M.Z (2011). Indian J Pharm Sci..

[19] Singh A (2022). Pharmaceutics..

[20] Chopra A (2010). J Ayurveda Integr Med..

[21] Nandan A (2023). Front Pharmacol..

[22] Balkrishna A (2020). Biomolecules..

[23] Di-Sotto A (2020). Vaccines (Basel)..

[24] Patel M.V (2011). Ayu..

